# The association between the interval of radioiodine treatment and treatment response, and side effects in patients with lung metastases from differentiated thyroid cancer

**DOI:** 10.3389/fendo.2023.1117001

**Published:** 2023-05-31

**Authors:** Hongxi Wang, Lei Shi, Rui Huang, Bin Liu, Rong Tian

**Affiliations:** ^1^ Department of Nuclear Medicine, West China Hospital, Sichuan University, Chengdu, Sichuan, China; ^2^ Department of Nuclear Medicine, Chengdu Fifth People’s Hospital, Chengdu, Sichuan, China

**Keywords:** differentiated thyroid cancer, lung metastases, radioiodine, interval, side effect

## Abstract

**Objective:**

Repeat radioiodine (RAI) treatment has been widely implemented for RAI-avid lung metastases and is clinically effective for lung metastatic differentiated thyroid cancer (DTC). We aim to investigate the association between the interval of RAI treatment and short-term response, and the side effects in patients with lung metastases from DTC and to identify predictors for non-effective response to the next RAI treatment.

**Methods:**

A total of 282 course pairs from 91 patients were established and categorized into two groups by the interval of neighboring RAI treatment (<12 and ≥12 months), and the characteristics and treatment response between the two groups were compared. Multivariate logistic regression was used to identify predictors associated with treatment response. The side effects in the former course and the latter course were compared while taking into account the interval.

**Results:**

No significant difference was found between the two groups in treatment response in the latter course (p > 0.05). In the multivariate analysis, age ≥ 55 years (OR = 7.29, 95% CI = 1.66–33.35, p = 0.008), follicular thyroid cancer (OR = 5.00, 95% CI = 1.23–22.18, p = 0.027), and a second RAI treatment as the former course (OR = 4.77, 95% CI = 1.42–18.61, p = 0.016) were significantly associated with a non-effective response. There was no significant difference in the side effects in the former and latter courses between the two groups (p > 0.05).

**Conclusion:**

The interval of RAI treatment does not affect short-term response and side effects of DTC patients with RAI-avid lung metastases. It was feasible to defer repeat evaluation and treatment with an interval of at least 12 months to obtain an effective response and reduce the risk of side effects.

## Introduction

Approximately 10% of patients with differentiated thyroid cancer (DTC) have distant metastases at presentation or during follow-up, and their lungs are the most common organ of distant metastases, accounting for 70% of distant metastasis cases (1, 2). DTC patients with lung metastases have a relatively poorer prognosis than those without metastases, with a median survival of <10 years (3). In the case of lung metastasis of DTC and continuous iodine uptake at the metastatic lesion, radioiodine (RAI) treatment is one of the most effective treatments (4).

No consensus is available to identify the interval of repeat RAI treatment for lung metastatic DTC. A wide selection of treatment intervals ranging from 3 to 12 months was largely based on expert experience (5–9), which has posed challenges to clinicians when developing RAI treatment strategies for patients with RAI-avid lung metastatic DTC. The American Thyroid Association (ATA) (5) recommended that RAI-avid micrometastases (<2 mm) should be treated with repeat RAI every 6–12 months as long as they respond clinically. For RAI-avid macronodular metastases (≥2 mm), RAI treatment was also recommended too, although how often to give RAI is a decision that must be individualized based on several factors, such as age, treatment response, other metastatic lesions, early and late side effects, and risk of second malignancies.

The incidence of side effects is associated with the cumulative dose of RAI and the number of RAI treatments (10, 11). Theoretically, on the premise of ensuring the RAI treatment response, longer intervals may decrease the frequency of interrupting thyroid-stimulating hormone (TSH) inhibition therapy and stimulating follicular cells and minimize the risk of side effects and secondary cancers, therefore improving prognosis and quality of life of patients (5, 6). There is an urgent need to discuss whether extended treatment intervals are clinically feasible and, if so, which patients can be treated with longer intervals to avoid overtreatment and reduce the incidence of side effects.

Moreover, with the cumulative dose of RAI increased, many patients with lung metastatic DTC did not respond, or they became refractory to RAI and had poorer prognoses (4, 12, 13). The targeted therapy was considered a feasible therapeutic strategy for those cases. However, there is no consensus on the indications for and optimal timing of targeted therapy (14). Thus, early identification of patients who do not respond to the next RAI treatment can help clinicians decide if and when to start targeted therapy.

Previous patient-based studies have demonstrated several variables associated with the efficacy of cumulative RAI treatment in patients with lung metastases from DTC (12, 15, 16). However, they hardly provide more reliable evidence for or against extending the interval between the neighboring RAI treatment, as well as predicting response to the next RAI treatment. Therefore, this course-based study aims to investigate the association between the interval of RAI treatment and short-term response, and side effects in patients with lung metastases from DTC and to identify predictors for non-effective response to the next RAI treatment.

## Methods

### Study participants

This retrospective analysis of totally thyroidectomized patients with RAI-avid lung metastatic DTC demonstrated by initial radioiodine post-therapeutic whole body scan (Rx-WBS) at West China Hospital, Sichuan University, from January 2009 to August 2018 was performed. All patients withdrew thyroid hormone medication and began a low iodine diet 3–4 weeks before initial RAI treatment, and the dose of RAI was 3.7–7.4 GBq at first treatment. Rx-WBS or RAI single-photon emission computed tomography/computed tomography (SPECT/CT) was performed 2–7 days after RAI administration. Levothyroxine was administered for thyroid hormone replacement or thyrotropin suppression 2–3 days after RAI administration (5, 17).

Follow-up was generally performed every 3–6 months following treatment, including measurements of TSH, thyroglobulin (Tg), anti-thyroglobulin antibody (TgAb), blood routine, blood biochemical examinations, neck ultrasonography, and chest CT. A diagnostic WBS (Dx-WBS) scan or positron emission tomography (PET/CT) was performed when necessary. According to these results, a treating physician would decide whether to perform repeat RAI treatment and the interval of repeat RAI treatment, in accordance with the ATA guidelines. The dose of repeat RAI treatment was 7.4 GBq. The RAI treatment was terminated once RAI-refractory DTC was diagnosed, patients did not benefit from RAI, or significant side effects occurred.

### Establishment of eligible course pairs

A course pair was established by neighboring two treatments (the former course and the latter course). The enrollment criteria of course pairs were RAI-avid lung metastatic DTC in patients who underwent at least three RAI treatments. The exclusion criteria were as follows: 1) course pairs interfered with by other distant metastases or concomitant malignancies; 2) course pairs interfered with by other therapeutics (pulmonary lobectomy, radiotherapy, and targeted therapy); 3) TSH before the RAI administration was <30 mIU/L or TgAb was > 60.0 IU/ml; 4) course pairs with an interval >24 months because of poor compliance; and 5) course pairs with missing treatment data. Eligible course pairs were then categorized into two groups according to the interval between courses: 1) <12 months and 2) ≥12 months.

### Serologic examinations and computed tomography scan

TSH, Tg, and TgAb levels were measured with the same high-sensitivity electrochemiluminescence immunoassay in the same laboratory. The assay used was the Roche Elecsys 2010 system (Roche Diagnostics GmbH, Mannheim, Germany), which was calibrated against the CRM‐457 standard, with a sensitivity of 0.04 ng/ml and a reference range of 0.5–55 ng/ml. Quality control was ensured by assaying two levels of control sera in each series and by reassessing all sera showing a coefficient of variation exceeding 10%.

A chest CT scan without iodinated contrast was performed extending from the lung apex to the adrenal glands at full inspiration just before RAI administration. Patients underwent scanning with a multidetector CT scanner (Somatom Definition Flash, Siemens Healthcare, Erlangen, Germany) with the following parameters: tube voltage, 100 keV; tube current, 100 mAs; thickness, 1 mm; interval, 1 mm; pitch, 1.2.

### Treatment response evaluation

The biochemical response was evaluated in the latter course by the comparison of stimulated Tg (s-Tg) just before RAI administration at two neighboring courses. Δs-Tg% (change rate of s-Tg) was defined as follows: Δs-Tg% = [s-Tg (the latter course) − s-Tg (the former course)]/s-Tg (the former course) × 100%. The following standards were used to determine the categorization of biochemical response (18, 19): s-Tg was <1 μg/L or Tg during TSH suppression was <0.2 μg/L in the absence of structural or functional evidence indicated complete remission (CR), Δs-Tg% ≤ −25% indicated partial remission (PR), Δs-Tg% > −25.0% and ≤ 25.0% indicated stable disease (SD), and Δs-Tg% > 25.0% indicated progressive disease (PD). Meanwhile, the structural response in the latter course was categorized as structural CR, PR, SD, and PD, according to the Response Evaluation Criteria in Solid Tumors (RECIST) 1.1-like criteria (20, 21). Therefore, the overall treatment response in the latter course was determined to be an effective response if SD or remission was obtained according to biochemical and structural response; if either of biochemical response or structural response assessment indicated PD, the treatment response was determined to be a non-effective response.

To investigate whether the association between interval and treatment response could be modified by RAI treatment times, we performed a stratified analysis by RAI treatment times of the former course (second, third and fourth, and fifth and more).

### Side effect evaluation

Side effects in the former courses and latter courses were compared taking into account a stratification of intervals. Side effects were defined as follows: 1) bone marrow suppression as a result of a reduction in white blood cell (WBC) count and platelets (PLT); 2) hypocalcemia presented as decreased serum calcium (Ca); 3) renal dysfunction presented as increased serum creatinine (Cr); 4) liver dysfunction as a result of an increase in serum glutamic oxaloacetic transaminase (AST), glutamic pyruvic transaminase (ALT), and AST/ALT ratio; 5) second primary malignant defined as a malignancy with non-thyroidal cancer metastasis occurring at least 12 months after RAI treatment. According to the National Cancer Institute Common Toxi<cp>city Criteria (NCI-CTC 2.0) (22), grade 0 represented a non-side effect, and the severity of side effects was divided into four grades: grade 1, grade 2, grade 3, and grade 4 (**Supplementary Table 1**).

### Statistical analysis

Continuous variables are expressed as (standard deviations [SDs]) or the medians (interquartile ranges [IQRs]), and categorical variables are presented as frequencies (percentages). The independent sample *t*-test, chi-square test, Fisher’s exact test, or Wilcoxon test was used to compare the characteristics and treatment response between the two groups with different intervals. Multivariate logistic regression analysis was used to identify predictors associated with non-effective responses. The Cochran–Mantel–Haenszel test was used to compare side effects in the former courses and latter courses taking into account a stratification of intervals. All the analyses were conducted in R software (version 4.0). A two-sided p-value of <0.05 was considered statistically significant.

## Results

### Characteristics of participants and course pairs

**Figure 1:**
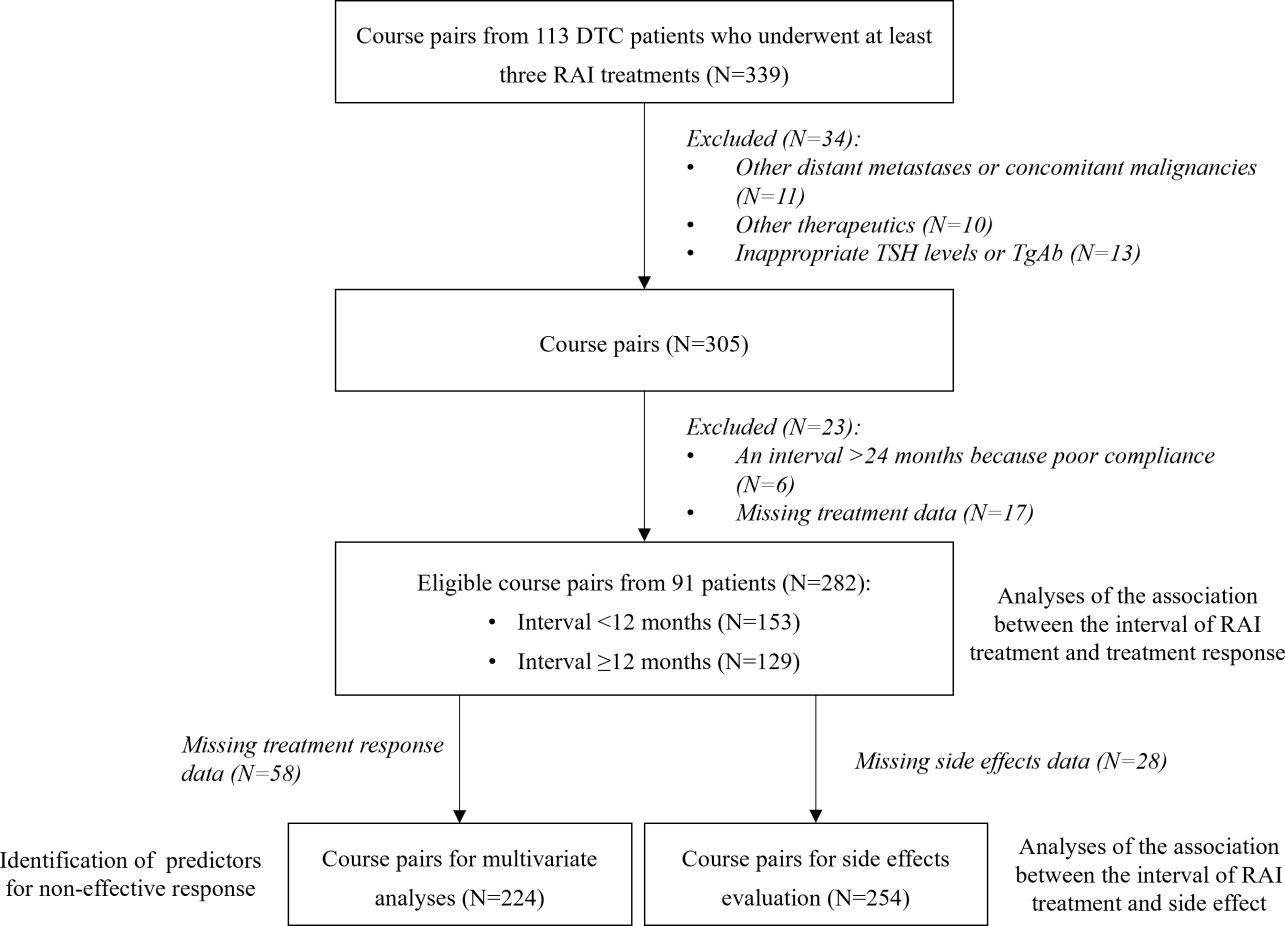
Flowchart of the study. DTC, differentiated thyroid cancer; TSH, thyroid-stimulating hormone; TgAb, anti-thyroglobulin antibody; RAI, radioiodine.

A total of 339 course pairs were enrolled from 113 patients. The following were excluded: A total of 339 course pairs were enrolled from 113 patients. The following were excluded: 11 course pairs interfered by combined or other distant metastasis and cancer beyond the thyroid, 10 course pairs interfered by other therapeutics, 13 course pairs with inappropriate TSH levels or elevated TgAb, 6 course pairs with intervals > 24 months because of poor compliance, and 17 course pairs with missing treatment data. Finally, a total of 282 eligible course pairs from 91 patients were established (**Figure 1**).

Of 282 course pairs from 91 patients (**Table 1**), the median age at treatment was 37.0 years. The former course was the first treatment, second treatment, third and fourth treatment, and fifth treatment and more in 90 (31.9%), 91 (32.3%), 73 (25.9%), and 28 (9.9%) course pairs, respectively. The median of s-Tg in the former course was 240.0 ng/ml. The cumulative RAI dose in the former course was ≥22.2 GBq at 188 former courses (66.7%). RAI-non-avid metastases concurrent with RAI-avid metastases were observed on Rx-WBS in the 17 former courses (6.0%).

**Table 1:** Characteristics of participants and course pairs.

Characteristics	No. (%)	Course pairs
	Patients	
Total	91	282
Age, median (IQR), years^a^	35 (23)	37 (23)
Sex		
Male	24 (26.4)	91 (32.3)
Female	67 (73.6)	191 (67.7)
Histology		
Classic PTC	71 (78.0)	225 (79.8)
FVPTC	13 (14.3)	36 (12.8)
FTC	7 (8.7)	21 (7.4)
T stage^b^		
T1	5 (5.5)	11 (3.9)
T2	6 (6.5)	20 (7.1)
T3	20 (22.0)	62 (22.0)
T4	36 (39.6)	100 (35.5)
Tx	24 (26.4)	89 (31.5)
N stage^b^		
N0	2 (2.2)	6 (2.1)
N1a	3 (3.3)	12 (4.3)
N1b	83 (91.2)	257 (91.1)
Nx	3 (3.3)	7 (2.5)
Treatment time		
3	48 (52.7)	NA
4 to 5	23 (25.3)	NA
≥6	20 (22.0)	NA
Size of metastatic lesion (mm)		
≤10	72 (79.1)	NA
>10	19 (20.9)	NA
Cumulative RAI dose, median (IQR), GBq	22.2 (16.6)	NA
Treatment time of former course		
First	NA	90 (31.9)
Second	NA	91 (32.3)
Third and fourth	NA	73 (25.9)
Fifth and more	NA	28 (9.9)
s-Tg in former course, median (IQR), ng/ml	NA	240.0 (720.0)
RAI-non-avid metastases concurrent with RAI-avid metastases on Rx-WBS in former course		
Yes	NA	17 (6.0)
No	NA	265 (94.0)
Cumulative RAI dose in former course (GBq)		
<22.2	NA	188 (66.7)
≥22.2	NA	94 (33.3)

PTC, papillary thyroid cancer; FVPTC, follicular variant of papillary thyroid cancer; FTC, follicular thyroid cancer; s-Tg, stimulated thyroglobulin; RAI, radioiodine; Rx-WBS, radioiodine post therapeutic whole body scan; NA, not applicable.

^a^Age was present as age at diagnosis in participants, and age in former course in course pairs.

^b^TNM staging was determined by 8th American Joint Cancer Committee Tumor-Node-Metastasis stage system.

### Comparison of characteristics and treatment response between course pairs with different intervals

As shown in **Table 2**, the intervals of a total of 153 course pairs were <12 months with a median of 6.3 months, and the intervals of 129 course pairs were ≥12 months with a median of 17.1 months. In general, the first treatment as a former course was more common in the group with an interval of <12 months, and the second or more treatment as a former course was more common in the group with an interval of ≥12 months (p = 0.001). The median of s-Tg in the former course in the <12 months interval group was higher than that in the ≥12 months interval group (290.0 and 180.0 ng/ml, p = 0.036). A higher cumulative RAI dose (≥22.2 GBq) was more common in the group with an interval of ≥12 months than in the group with an interval of <12 months (54.3% *vs.* 15.7%, p = 0.001). No significant difference was found between the two groups in age, sex, histology, T stage, N stage, or lesion uptake on Rx-WBS (p > 0.05).

**Table 2:** The comparison of characteristics and treatment responses between course pairs with different intervals.

Characteristics and treatment responses	No. (%)		p-Value
	<12 months	≥12 months	
Total	153	129	
Age in former course, median (IQR), years	37 (22)	36 (25)	0.680
Sex			0.586
Male	52 (34.0)	39 (30.2)	
Female	101 (66.0)	90 (69.8)	
Histology			0.814
Classic PTC	123 (80.4)	102 (79.1)	
FVPTC	20 (13.1)	16 (12.4)	
FTC	10 (6.5)	11 (8.5)	
T stage			0.463
T1	6 (3.9)	5 (43.9)	
T2	12 (7.8)	8 (6.2)	
T3	36 (23.5)	26 (20.2)	
T4	58 (37.9)	42 (32.6)	
Tx	41 (26.8)	48 (37.2)	
N stage			0.394
N0	4 (2.6)	2 (1.6)	
N1a	8 (5.2)	4 (3.1)	
N1b	139 (90.8)	118 (91.5)	
Nx	2 (1.3)	5 (3.9)	
Treatment time of former course			0.001
First	84 (54.9)	6 (4.7)	
Second	45 (29.4)	46 (35.7)	
Third and fourth	22 (14.4)	51 (39.5)	
Fifth and more	2 (1.3)	26 (20.2)	
s-Tg in former course, median (IQR), ng/ml	290.0 (880.0)	180.0 (420.0)	0.036
Cumulative RAI dose in former course (GBq)			0.001
<22.2	129 (84.3)	59 (45.7)	
≥22.2	24 (15.7)	70 (54.3)	
RAI-non-avid metastases concurrent with RAI-avid metastases on Rx-WBS in former course			0.716
Yes	8 (5.2)	9 (7.0)	
No	145 (94.8)	120 (93.0)	
Δs-Tg% in latter course, median (IQR), %	−35.6 (45.1)	−32.4 (36.8)	0.271
Biochemical response in latter course			
CR	1 (0.7)	3 (2.3)	0.169
PR	88 (57.5)	76 (58.9)	
SD	51 (33.3)	32 (24.8)	
PD	13 (8.5)	18 (14.0)	
Structural response in latter course			0.569
CR	13 (8.5)	14 (10.9)	
PR	97 (63.4)	87 (67.4)	
SD	3 (2.0)	3 (2.3)	
PD	40 (26.1)	25 (19.4)	
Treatment response in latter course			0.161
Effective	102 (66.7)	90 (69.8)	
Non-effective	14 (9.2)	18 (14.0)	
Missing	37 (24.2)	21 (16.3)	

PTC, papillary thyroid cancer; FVPTC, follicular variant of papillary thyroid cancer; FTC, follicular thyroid cancer; s-Tg, stimulated thyroglobulin; RAI, radioiodine; Rx-WBS, radioiodine post therapeutic whole body scan; CR, complete remission; PR, partial remission; SD, stable disease; PD, progressive disease.

**Figure 2:**
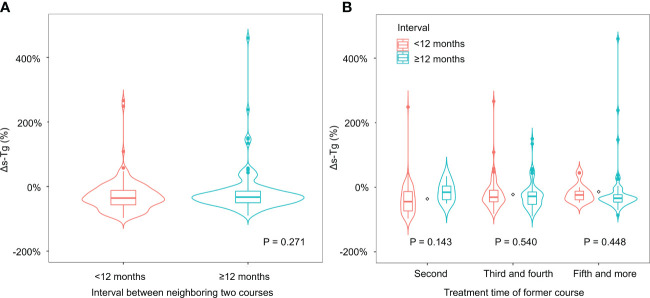
**(A)** The Δs-Tg% of course pairs with different intervals. **(B)** The Δs-Tg% of course pairs with different intervals in subgroups.

No significant difference was found between the two groups in Δs-Tg% (**Figure 2A**, p = 0.271) or biochemical response in the latter time point (p = 0.169). Similarly, no significant difference was found between the two groups in structural response (p = 0.569) or overall treatment response (p = 0.161) in the latter course. In addition, we obtained similar results across different groups of RAI treatment time (**Supplementary Table 2**; **Figure 2B**).

### Predictors of non-effective response in multivariate analyses

The multivariate analysis was performed among 224 course pairs, as course pairs with missing treatment response data were removed (**Figure 1**). A non-effective response was obtained in 32 courses (14.3%), and patients with age ≥ 55 years in the former and latter were more likely to obtain a non-effective response in the latter course (OR = 7.29, 95% CI = 1.66–33.35, p = 0.008), compared with patients with age < 55 years. Patients with follicular thyroid cancer (FTC) were more likely to obtain non-effective responses (OR = 5.00, 95% CI = 1.23–22.18, p = 0.027) than patients with papillary thyroid cancer (PTC). Non-effective response to the second treatment was more common than non-effective response to the first treatment (OR = 4.77, 95% CI = 1.42–18.61, p = 0.016). The interval was not associated with a non-effective response (p = 0.446) (**Table 3**).

**Table 3:** Multivariate logistic regression analysis of predictors of non-effective response.

Predictors	B	Wald	OR (95% CI)	p
(Intercept)	−18.97	0.00		
Age				
<55				
≥55	1.98	6.97	7.29 (1.66–33.35)	0.008
Sex				
Male				
Female	0.88	2.68	2.42 (0.88–7.50)	0.102
T stage				
T1–T3				
T4	0.33	0.31	1.40 (0.44–4.81)	0.573
Tx	0.53	0.75	1.70 (0.52–5.95)	0.388
N stage				
N0				
N1a+N1b	16.41	0.00	13431948.39 (0–NA)	0.997
Nx	13.87	0.00	1059652.34 (0–NA)	0.998
Histology				
PTC				
FVPTC	−16.94	0.00	0 (0–Inf)	0.988
FTC	1.61	4.88	5.00 (1.23–22.18)	0.027
Interval				
<12 months				
≥12 month	−0.401	0.58	0.67 (0.24–1.88)	0.446
Treatment time of former course				
First				
Second	1.56	5.85	4.77 (1.42–18.61)	0.016
Third and fourth	0.87	0.53	2.39 (0.18–22.25)	0.467
Fifth and more	1.84	1.69	6.28 (0.34–95.35)	0.193
Cumulative RAI dose in former course, GBq				
<22.2				
≥22.2	0.26	0.06	1.30 (0.18–14.61)	0.811
RAI-non-avid metastases concurrent with RAI-avid metastases on Rx-WBS in former course				
Yes				
No	−1.51	4.13	0.22 (0.05–1.00)	0.052
s-Tg in former course, ng/ml^a^				
≤242.2				
>242.2	0.18	0.16	1.20 (0.50–2.93)	0.691

OR, odds ratio; CI, confidence interval; PTC, papillary thyroid cancer; FVPTC, follicular variant of papillary thyroid cancer; FTC, follicular thyroid cancer; s-Tg, stimulated thyroglobulin; RAI, radioiodine; Rx-WBS, radioiodine post therapeutic whole body scan; CR, complete remission; PR, partial remission; SD, stable disease; PD, progressive disease.

^a^The cut-off was settled at the median of s-Tg.

### Comparison of side effects in the former course and the latter course between course pairs with different intervals

The analysis was performed among 254 course pairs, as course pairs with missing side effects data were removed (**Figure 1**). The distribution of side effects in the latter course is shown in **Table 4**. There was no significant difference in the distribution of WBC, PLT, Ca, Cr, AST, ALT, and AST/ALT ratio in the former and latter courses between the group with an interval of <12 months and the group with an interval of ≥12 months (p > 0.05) (**Table 5**). No patient developed a second primary malignancy during the follow-up period.

**Table 4:** The distribution of side effects in the latter course.

Side effects	Reference range	No. (%)
		Latter course (N = 254)
Bone marrow suppression (WBC)		
Grade 0	≥4.0 10^9^/L	223 (87.8)
Grade 1	3.0–4.0 10^9^/L	26 (10.2)
Grade 2	2.0–3.0 10^9^/L	5 (2.0)
Bone marrow suppression (PLT)		
Grade 0	≥100.0 10^9^/L	238 (93.7)
Grade 1	75.0–100.0 10^9^/L	13 (5.1)
Grade 2	50.0–75.0 10^9^/L	3 (1.2)
Hypocalcemia (Ca)		
Grade 0	≥2.1 mmol/L	163 (64.2)
Grade 1	2.0–2.1 mmol/L	45 (17.7)
Grade 2	1.75–2.0 mmol/L	24 (9.5)
Grade 3	1.5–1.75 mmol/L	16 (6.3)
Grade 4	<1.5 mmol/L	6 (2.4)
Renal dysfunction (Cr)		
Grade 0	≤110.0 μmol/L	254 (100.0)
Liver dysfunction (AST)		
Grade 0	<35.0 IU/L	210 (82.7)
Grade 1	35.0–87.5 IU/L	43 (16.9)
Grade 2	87.5–175.0 IU/L	1 (0.4)
Liver dysfunction (ALT)		
Grade 0	<40.0 IU/L	212 (83.5)
Grade 1	40.0–100.0 IU/L	40 (15.7)
Grade 2	100.0–200.0 IU/L	2 (0.8)
Liver dysfunction (AST/ALT ratio)		
Grade 0	0.5–2.5	240 (94.5)
Grade 1	2.5–5.0	1 (0.4)
Grade 2	5.0–20.0	13 (5.1)

WBC, white blood cell; PLT, platelets; Ca, serum calcium; Cr, serum creatinine; AST, glutamic oxaloacetic transaminase; ALT, glutamic pyruvic transaminase.

**Table 5:** Comparison of side effects in the former and latter courses between the group with an interval of <12 months and the group with an interval of ≥12 months.

Side effects	Former course (N = 254)	Latter course (N = 254)		p-Value
		Grade 0	Grade 1–4	
Bone marrow suppression (WBC)				0.543
<12 months (N = 136)	Grade 0	107	13	
	Grade 1–2	8	8	
≥12 months (N = 118)	Grade 0	101	3	
	Grade 1–2	7	7	
Bone marrow suppression (PLT)				0.693
<12 months (N = 136)	Grade 0	129	2	
	Grade 1–2	4	1	
≥12 months (N = 118)	Grade 0	100	8	
	Grade 1–2	5	5	
Hypocalcemia (Ca)				0.210
<12 months (N = 136)	Grade 0	86	13	
	Grade 1–4	9	28	
≥12 months (N = 118)	Grade 0	53	17	
	Grade 1–4	15	33	
Renal dysfunction (Cr)				NA
<12 months (N = 136)	Grade 0	136	0	
	Grade 1–4	0	0	
≥12 months (N = 118)	Grade 0	118	0	
	Grade 1–4	0	0	
Liver dysfunction (AST)				0.442
<12 months (N = 136)	Grade 0	102	7	
	Grade 1–2	13	14	
≥12 months (N = 118)	Grade 0	82	16	
	Grade 1–2	13	7	
Liver dysfunction (ALT)				0.891
<12 months (N = 136)	Grade 0	101	10	
	Grade 1–2	14	11	
≥12 months (N = 118)	Grade 0	85	12	
	Grade 1–2	12	9	
Liver dysfunction (AST/ALT ratio)				0.712
<12 months (N = 136)	Grade 0	130	2	
	Grade 1–2	2	2	
≥12 months (N = 118)	Grade 0	106	4	
	Grade 1–2	2	6	

NA, not apply; WBC, white blood cell; PLT, platelets; Ca, serum calcium; Cr, serum creatinine; AST, glutamic oxaloacetic transaminase; ALT, glutamic pyruvic transaminase.

## Discussion

Repeat RAI treatment has been widely implemented for RAI-avid and clinically effective for lung metastatic DTC, and RAI treatment response has been recognized as an important predictor of long-term prognosis and survival in DTC patients. However, whether the length of the interval between RAI treatment was associated with response or side effects is unclear. To our knowledge, this is the first study to investigate the association between the interval, short-term treatment response, and side effects in patients with RAI-avid lung metastatic DTC.

A precise definition of an effective response to RAI treatment is not feasible given the wide variation in disease presentation and response to RAI treatment. A meaningful response is usually associated with a significant reduction in serum Tg and/or in the size or rate of growth of metastases or structurally apparent disease (5). In the present study, along with biochemical and structural remission, we categorized biochemical and structural stabilization into effective response because a majority of patients with lung metastases will not have a complete response and will take years to see the full response of RAI. Thus, prospective studies are needed to illustrate whether biochemical and structural stable disease truly represents an effective response to RAI treatment.

In this study, an interval of ≥12 months led to a similar treatment response to RAI treatment, which is a longer interval than the usually reported interval (<12 months), indicating that in DTC patients with RAI-avid lung metastatic, it is feasible to postpone repeat evaluation and treatment at intervals of at least 12 months until a complete response is obtained. In addition, treatment time and cumulative dose were important factors when determining the interval in clinical practice. In course pairs with an interval of ≥12 months (median, 17.1 months), we found that second or more RAI treatment as a former course and a higher cumulative RAI dose were more common than in course pairs with an interval of <12 m (median, 6.3 months). Therefore, course pairs were categorized into three subgroups according to treatment times of the former course (second, third and fourth, and fifth and more) in the stratified analysis, and the results were similar to those of the main analysis.

Our study provided real-world evidence for previous studies and expert consensus on repeat RAI treatment intervals. It has been reported that the maximal clinical response from RAI treatment may not be reached for up to 15–18 months (23). In children with lung metastatic DTC, sustained improvement in Tg levels can be found several years after discontinuation of RAI treatment (24). Therefore, the ATA guideline suggested that for serologic progression, waiting at least 12 months would better establish a trend to ensure that elevated Tg or TgAb levels are not spurious or due to RAI-induced tumor destruction (6).

We found that age ≥ 55 years and FTC were significantly associated with non-effective responses, which were consistent with previous findings (25–27). Meanwhile, a second RAI treatment as the former course was a predictor of non-effective response; this could be partly attributed to more than half of the patients (48 cases, 52.7%) who underwent three RAI treatments in this study. Therefore, for patients with a high risk of non-effective response, further re-evaluation and treatment would be warranted balancing cumulative dose, side effects, and patient desires as long as it has been more than 12 months since the former RAI treatment. Moreover, there was a discrepancy compared to previous studies; another course-based study (18) identified the change rate of TSH-suppressed Tg (DTgon%) of 8.1 and maximum target/background ratio on WBS (T/Bmax) of 25.3% as predictors for biochemical response to next RAI treatment in DTC lung metastasis. The reasons for this discrepancy may be because of heterogeneity in the response assessment criteria and the included variable in the analysis.

In this study, we compared the distribution of side effects in the former courses and latter courses, including bone marrow suppression, liver dysfunction, hypocalcemia, renal dysfunction, and second primary malignant, while taking into account a stratification of interval. The results illustrated that interval was not associated with the distribution of side effects in the former courses and latter courses. Previous studies have proposed an activity–response relationship in which the risk of side effects tends to increase above a cumulative dose of 3.1–11.1 GBq (5). Acute bone marrow suppression may occur, but hematologic parameters usually normalize within 60 days after RAI administration. The kidneys are the main way of iodine excretion, radiation to the bone marrow can be impacted by renal function, and renal impairment significantly reduces RAI excretion (28). Serum calcium decreased 5 days after RAI treatment and recovered 6 weeks after treatment (29). Liver function decreased significantly after RAI ablation and then showed a recovery trend after multiple treatments (30). Therefore, it is essential to allow for the recovery of side effects between RAI treatments, and a longer interval between RAI treatments may minimize the risk of late side effects, which needs to be further elucidated based on long-term data.

Our study had some limitations. First, the retrospective design might have resulted in selection bias. Our analyses were mainly based on data from patients with younger age, metastasis at diagnosis, PTC, and a lesion size ≤10 mm. Thus, our results should be considered with caution for specific patients with significantly different clinical features and prognoses. Second, some side effects were not analyzed, such as salivary gland and gonadal dysfunction. Third, the interval of treatment for each patient was individually managed in real-world clinical practice, and the influence of interval on long-term treatment response and side effects needs to be confirmed in prospective randomized controlled studies.

## Conclusion

The interval of RAI treatment does not affect the short-term response and side effects of DTC patients with RAI-avid lung metastases. In those patients, it was feasible to defer repeat evaluation and treatment with an interval of at least 12 months to obtain an effective response and reduce the risk of side effects. In addition, older age, FTC, and a second RAI treatment as a former course were independent predictors for a non-effective response. For patients with a high risk of non-effective response, further re-evaluation and treatment would be warranted balancing cumulative dose, side effects, and patient desires, as long as it has been more than 12 months since the former RAI treatment.

## Data availability statement

The data analyzed in this study is subject to the following licenses/restrictions: The datasets generated during and/or analyzed during the current study are available from the corresponding author upon reasonable request. Requests to access these datasets should be directed to wanghxnuclear@163.com.

## Ethics statement

The study protocol was approved by the Institutional Research Ethics Committee of West China Hospital of Sichuan University (# 2020678), and the requirement of written informed consent was waived.

## Author contributions

RH and RT contributed to the study’s conception and design. Material preparation, data collection, and analysis were performed by HW and LS. The first draft of the manuscript was written by HW and LS. All authors commented on previous versions of the manuscript. All authors contributed to the article and approved the submitted version.
