# Supplementary material for: The association between the interval of radioiodine treatment and treatment response, and side effects in patients with lung metastases from differentiated thyroid cancer
Source: Front Endocrinol (Lausanne). 2023 May 31;14:1117001. doi: 10.3389/fendo.2023.1117001 (PMC10264775; doi:10.3389/fendo.2023.1117001)
Supplement: Supplementary file 1 [file DataSheet_1.docx]

SUPPLEMENTARY TABLE 1 The National Cancer Institute Common Toxicity Criteria (NCI-CTC 2.0).

| Side effects | Grade 0: normal | Grade 1: mild | Grade 2: moderate | Grade 3: severe | Grade 4: life-threatening |
| --- | --- | --- | --- | --- | --- |
| Bone marrow suppression |  |  |  |  |  |
| White blood cell (WBC), 10^9^ /L | 3.5 - 9.5 | 3.5 - 3.0 | 2.0 - 3.0 | 1.0 - 2.0 | <1.0 |
| Platelets (PLT), 10^9^ /L | 100.0 - 300.0 | 100.0 - 75.0 | 50.0 - 75.0 | 10.0 - 50.0 | <10.0 |
| Liver dysfunction |  |  |  |  |  |
| Glutamic oxaloacetic transaminase (AST), IU/L | ≤ 35.0 | 35.0 - 87.5 | 87.5 - 175.0 | 175.0 - 700.0 | > 700 |
| Glutamic pyruvic transaminase (ALT), IU/L | ≤ 40.0 | 40.0 - 100 | 100.0 - 200.0 | 200.0 - 800.0 | > 800 |
| AST/ALT ratio^a^ | 0.5 - 2.5 | 2.5 - 5.0 | 5.0 - 20.0 | > 20.0 | NA |
| Hypocalcemia |  |  |  |  |  |
| Calcium (Ca), mmol/L | 2.1 - 2.7 | 2.0 - 2.1 | 1.75 - 2.0 | 1.5 - 1.75 | <1.5 |
| Renal dysfunction |  |  |  |  |  |
| Creatinine (Cr), umol/L | 37.0 - 110.0 | 110.0 - 165.0 | 165.0 - 330.0 | 330.0 - 660.0 | > 660.0 |

NA, not apply.

^a^ AST/ALT ratio was evaluated based on empiric data.

SUPPLEMENTARY TABLE 2 Comparison of characteristics and treatment response between course pairs with different intervals in subgroup analyses.

| Characteristics and treatment response | No. (%) | | | | | | | | | | |
| --- | --- | --- | --- | --- | --- | --- | --- | --- | --- | --- | --- |
|  | Subgroup 1: Second (N=90) | | |  | Subgroup 2: Third to Fourth (N=133) | | |  | Subgroup 3: Fifth and more (N=59) | | |
|  | <12 months | ≥12 months | P value |  | <12 months | ≥12 months | P value |  | <12 months | ≥12 months | P value |
| Total | 84 (93.3) | 6 (6.7) |  |  | 60 (45.1) | 73 (54.9) |  |  | 9 (15.3) | 50 (84.7) |  |
| Age, median (IQR), years | 37 (23) | 36 (24) | 0.981 |  | 38 (22) | 36 (25) | 0.160 |  | 33 (16) | 41 (21) | 0.183 |
| Sex |  |  | 0.923 |  |  |  | 0.134 |  |  |  | 0.106 |
| Male | 23 (27.4) | 1 (16.7) |  |  | 22 (36.7) | 17 (23.3) |  |  | 7 (78.8) | 21 (42.0) |  |
| Female | 61 (72.6) | 5 (83.3) |  |  | 38 (63.3) | 56 (76.7) |  |  | 2 (22.2) | 29 (58.0) |  |
| Histology |  |  | 0.447 |  |  |  | 0.837 |  |  |  | 0.548 |
| Classic PTC | 65 (77.4) | 5 (83.3) |  |  | 50 (83.3) | 58 (79.5) |  |  | 8 (88.9) | 39 (78.0) |  |
| FVPTC | 13 (15.5) | 0 (0.0) |  |  | 7 (11.7) | 10 (13.7) |  |  | 0 (0.0) | 6 (12.0) |  |
| FTC | 6 (7.1) | 1 (16.7) |  |  | 3 (5.0) | 5 (6.8) |  |  | 1 (11.1) | 5 (10.0) |  |
| T stage |  |  | 0.235 |  |  |  | 0.564 |  |  |  | 0.783 |
| T1 | 5 (6.0) | 0 (0.0) |  |  | 1 (1.7) | 5 (6.8) |  |  | 0 (0.0) | 0 (0.0) |  |
| T2 | 6 (7.1) | 0 (0.0) |  |  | 5 (8.3) | 4 (5.5) |  |  | 1 (11.1) | 4 (8.0) |  |
| T3 | 19 (22.6) | 1 (16.7) |  |  | 14 (23.3) | 13 (17.8) |  |  | 3 (33.3) | 12 (24.0) |  |
| T4 | 34 (40.5) | 1 (16.7) |  |  | 23 (38.3) | 28 (38.4) |  |  | 1 (11.1) | 13 (26.0) |  |
| Tx | 20 (23.8) | 4 (66.7) |  |  | 17 (28.3) | 23 (31.5) |  |  | 4 (44.4) | 21 (42.0) |  |
| N stage |  |  | 0.281 |  |  |  | 0.090 |  |  |  | 0.786 |
| N0 | 2 (2.4) | 0 (0.0) |  |  | 2 (3.3) | 1 (1.4) |  |  | 0 (0.0) | 1 (2.0) |  |
| N1a | 3 (3.6) | 0 (0.0) |  |  | 4 (6.7) | 1 (1.4) |  |  | 1 (11.1) | 3 (6.0) |  |
| N1b | 77 (91.7) | 5 (83.3) |  |  | 54 (90.0) | 67 (91.8) |  |  | 8 (88.9) | 46 (92.0) |  |
| Nx | 2 (2.4) | 1 (16.7) |  |  | 0 (0.0) | 4 (5.5) |  |  | 0 (0.0) | 0 (0.0) |  |
| s-Tg at former course, median (IQR), ng/ml | 420.0 (760.0) | 180.0 (730.0) | 0.461 |  | 170.0 (890.0) | 180.0 (490.0) | 0.648 |  | 91.0 (3100.0) | 220.0 (390.0) | 0.792 |
| Cumulative RAI dose at former course, GBq |  |  | 1.000 |  |  |  | 0.730 |  |  |  | 1.000 |
| <22.2 | 83 (98.8) | 6 (100.0) |  |  | 46 (76.7) | 53 (72.6) |  |  | 0 (0.0) | 0 (0.0) |  |
| ≥22.2 | 1 (1.2) | 0 (0.0) |  |  | 14 (23.3) | 20 (27.4) |  |  | 9 (100.0) | 50 (100.0) |  |
| RAI-nonavid metastases concurrent with RAI-avid metastases on Rx-WBS at former course |  |  | 1.000 |  |  |  | 0.578 |  |  |  | 1.000 |
| Yes | 4 (4.8) | 0 (0.0) |  |  | 4 (6.7) | 8 (11.0) |  |  | 0 (0.0) | 1 (2.0) |  |
| No | 80 (95.2) | 6 (100.0) |  |  | 56 (93.3) | 65 (89.0) |  |  | 9 (100.0) | 49 (98.0) |  |
| Δ s-Tg% at latter course, median (IQR), % | -42.7 (60.0) | -15.5 (42.2) | 0.143 |  | -30.8 (35.8) | -27.2 (37.7) | 0.540 |  | -23.7 (26.7) | -33.7 (31.7) | 0.448 |
| Biochemical response at latter course |  |  | 0.652 |  |  |  | 0.441 |  |  |  | 0.210 |
| CR | 1 (1.2) | 0 (0.0) |  |  | 0 (0.0) | 3 (4.1) |  |  | 0 (0.0) | 0 (0.0) |  |
| PR | 52 (61.9) | 3 (50.0) |  |  | 32 (53.3) | 39 (53.4) |  |  | 4 (44.4) | 34 (68.0) |  |
| SD | 27 (32.1) | 2 (33.3) |  |  | 20 (33.3) | 21 (28.8) |  |  | 4 (44.4) | 9 (18.0) |  |
| PD | 4 (4.8) | 1 (16.7) |  |  | 8 (13.3) | 10 (13.7) |  |  | 1 (11.1) | 7 (14.0) |  |
| Structural response at latter course |  |  | 0.234 |  |  |  | 0.064 |  |  |  | 0.888 |
| PR | 9 (10.7) | 0 (0.0) |  |  | 3 (5.0) | 11 (15.1) |  |  | 1 (11.1) | 3 (6.0) |  |
| SD | 56 (66.7) | 6 (100.0) |  |  | 36 (60.0) | 49 (67.1) |  |  | 5 (55.6) | 32 (64.0) |  |
| PD | 0 (0.0) | 0 (0.0) |  |  | 3 (5.0) | 2 (2.7) |  |  | 0 (0.0) | 1 (2.0) |  |
| Missing | 19 (22.6) | 0 (0.0) |  |  | 18 (30.0) | 11 (15.1) |  |  | 3 (33.3) | 14 (28.0) |  |
| Treatment response at latter course |  |  | 0.260 |  |  |  | 0.053 |  |  |  | 0.835 |
| Effective | 63 (75.0) | 5 (83.3) |  |  | 34 (56.7) | 54 (74.0) |  |  | 5 (55.6) | 31 (62.0) |  |
| Non-effective | 4 (4.8) | 1 (16.7) |  |  | 9 (15.0) | 10 (13.7) |  |  | 1 (11.1) | 7 (14.0) |  |
| Missing | 17 (20.2) | 0 (0.0) |  |  | 17 (28.3) | 9 (12.3) |  |  | 3 (33.3) | 12 (24.0) |  |

PTC, papillary thyroid cancer; FVPTC, follicular variant of papillary thyroid cancer; FTC, follicular thyroid cancer; s-Tg, stimulated thyroglobulin; RAI, radioiodine; Rx-WBS, radioiodine post therapeutic whole body scan; CR, complete remission; PR, partial remission; SD, stable disease; PD, progressive disease.
